# Targeting Cellular Trafficking of Fibroblast Growth Factor Receptors as a Strategy for Selective Cancer Treatment

**DOI:** 10.3390/jcm8010007

**Published:** 2018-12-20

**Authors:** Natalia Porębska, Marta Latko, Marika Kucińska, Małgorzata Zakrzewska, Jacek Otlewski, Łukasz Opaliński

**Affiliations:** Department of Protein Engineering, Faculty of Biotechnology, University of Wrocław, Joliot-Curie 14a, 50-383 Wroclaw, Poland; natalia.porebska2@uwr.edu.pl (N.P.); marta.latko2@uwr.edu.pl (M.L.); kucinska.marika@gmail.com (M.K.); malgorzata.zakrzewska@uwr.edu.pl (M.Z.); jacek.otlewski@uwr.edu.pl (J.O.)

**Keywords:** FGFR, signaling, cancer, protein transport, targeted therapy

## Abstract

Fibroblast growth factor receptors (FGFRs) in response to fibroblast growth factors (FGFs) transmit signals across the cell membrane, regulating important cellular processes, like differentiation, division, motility, and death. The aberrant activity of FGFRs is often observed in various diseases, especially in cancer. The uncontrolled FGFRs’ function may result from their overproduction, activating mutations, or generation of FGFRs’ fusion proteins. Besides their typical subcellular localization on the cell surface, FGFRs are often found inside the cells, in the nucleus and mitochondria. The intracellular pool of FGFRs utilizes different mechanisms to facilitate cancer cell survival and expansion. In this review, we summarize the current stage of knowledge about the role of FGFRs in oncogenic processes. We focused on the mechanisms of FGFRs’ cellular trafficking—internalization, nuclear translocation, and mitochondrial targeting, as well as their role in carcinogenesis. The subcellular sorting of FGFRs constitutes an attractive target for anti-cancer therapies. The blocking of FGFRs’ nuclear and mitochondrial translocation can lead to the inhibition of cancer invasion. Moreover, the endocytosis of FGFRs can serve as a tool for the efficient and highly selective delivery of drugs into cancer cells overproducing these receptors. Here, we provide up to date examples how the cellular sorting of FGFRs can be hijacked for selective cancer treatment.

## 1. Introduction

Receptor tyrosine kinases (RTKs) constitute a large family of integral plasma membrane proteins that, by binding to appropriate ligands, transmit signals across the cell membrane. RTKs regulate a number of pivotal cellular processes, including cell division, motility, differentiation, metabolism, and survival. In humans, the RTK family constitutes of 58 members that share a similar structure and mode of action [1]. RTKs are composed of an extracellular region responsible for ligand binding, a single transmembrane α-helix that embeds these receptors in the cellular membranes, and an intracellular region with a tyrosine kinase domain responsible for signal propagation [2]. In the majority of cases, RTKs are monomers in their inactive state, whereas ligand binding results in RTKs’ dimerization and activation, followed by the initiation of signaling cascades [3]. In addition, RTK-determined cell fate depends on the strength and duration of the signal propagation [4]. 

Because of their critical function for cell homeostasis, the activity of RTKs is under tight control at various levels [5]. Aberrant RTKs signaling is implicated in a number of severe human diseases, especially in cancer [6]. Versatile activating mechanisms are responsible for unbalanced, oncogenic RTKs signaling, including chromosomal rearrangements, gain of function mutations, gene amplifications, and autocrine activation [3,6]. Therefore, the dysregulated RTKs signaling has become an attractive target for the development of novel anticancer therapies [7,8,9]. 

In this review, we discuss the role of fibroblast growth factor receptors (FGFRs), a subfamily of RTKs, in cancer development. In particular, we focus on the subcellular trafficking of FGFRs and its implications in cancer treatment.

## 2. Fibroblast Growth Factor Receptors

### 2.1. Structure of FGFRs

**Figure 1:**
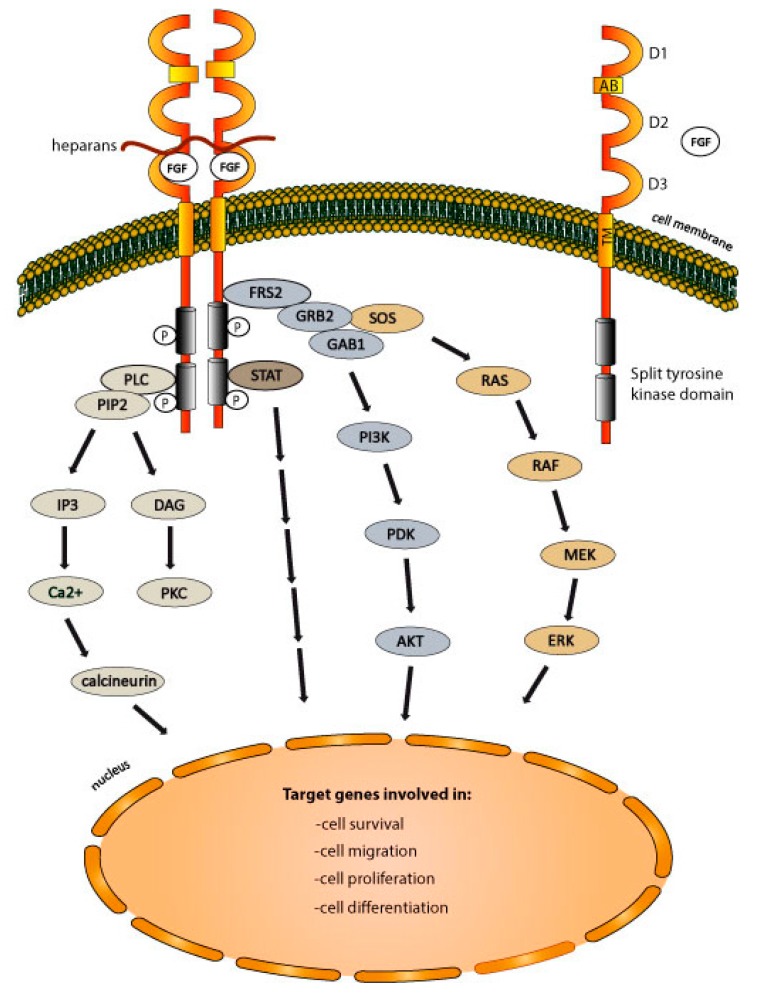
Fibroblast growth factor receptors (FGFRs)-dependent signaling pathways. Binding of FGFs stimulates FGFRs dimerization, leading to receptor autophosphorylation and to the recruitment of downstream signaling molecules that further propagate the signals (for details, see the main text of the manuscript).

FGFRs are integral membrane proteins with an overall structure typical for RTKs. Four major FGFR proteins have been identified, namely: FGFR1–FGFR4. FGFRs contain an extracellular region, a single transmembrane span, and an intracellular tyrosine kinase domain (Figure 1). The extracellular part of the FGFRs is composed of three immunoglobulin-like domains, D1, D2, and D3. The D1 domain prevents the receptor from autoactivation in the absence of appropriate ligands, and regulates the receptor affinity towards the FGFs [10,11,12,13]. The D1 and D2 domains are connected by a flexible linker rich in negatively charged residues, called acidic box (AB). The AB participates in FGFRs interaction with partner proteins and in receptors’ autoinhibition [10,14]. The D2 and D3 domains form the growth factor binding site. Additionally, the D2 domain contains a positively charged heparin binding region [15]. The single transmembrane α-helix ensures the subcellular localization of FGFRs and is involved in receptor dimerization [16,17]. The juxtamembrane region of FGFRs plays a regulatory role in signaling and modulates receptor dimerization [18,19,20]. The intracellular split tyrosine kinase domain is essential for the initiation of signaling cascades [21,22]. Interestingly, FGFRL1 (or FGFR5), a protein with domain organization resembling canonical FGFRs, was identified [23,24]. Similar to FGFRs, FGFRL1 contains an extracellular region composed of D1–D3 domains and a single transmembrane α-helix, but the intracellular region of FGFRL1 lacks tyrosine kinase domain typical for FGFRs [23,25]. The cytoplasmic part of FGFRL1 is composed of a short tail that has no kinase activity, however, it is still capable of recruiting signaling proteins, like SHP1 [26]. FGFRL1, besides participation in cellular signaling, is involved in cell–cell contacts and differentiation [27,28,29,30].

FGFRs are activated by fibroblast growth factors (FGFs), which belong to the following two major groups: canonical FGFs (FGF1–FGF10, FGF16–FGF18, FGF20, and FGF22) and endocrine FGFs (FGF15/19, FGF21, and FGF23). The members of the third group of FGFs, the intracellular FGFs (FGF11–FGF14), have no identified interaction with FGFRs, and thus are not able to stimulate FGFRs [21,22]. The specificity of the four FGFRs towards particular FGF ligands is determined by the D2–D3 domains, and the flexible linker connecting these regions. The splicing within the extracellular part of FGFR1–FGFR3 yields two major receptor variants, b and c, that display different ligand selectivity [31,32,33,34,35,36,37]. In contrast, FGFR4 is not processed by an alternative splicing within the ligand binding region [38]. It is noteworthy that FGFRs’ alternative splicing may lead to the generation of other receptor variants (e.g., lacking transmembrane region or produce secreted fragments of FGFRs) [39,40,41]. The distribution of alternatively spliced FGFRs displays tissue specificity that is relevant for coordinated organogenesis [21,22,40].

### 2.2. The Mechanism of FGFRs Activation

The current model of FGFRs’ activation states that in an inactive state, FGFRs are present on the cell membrane as monomers. FGF binding induces conformational changes within the receptor, causing receptor dimerization and the activation of tyrosine kinase domains (Figure 1) [37]. Interestingly, the spatial positioning of FGFRs in complexes with various FGFs differs, which may influence the strength, duration, and specificity of the propagated signals [11,37]. The canonical model of the ligand-induced FGFR dimerization was recently challenged by the observation that FGFRs may form active dimers in the absence of FGFs [13,42,43,44]. Ligand-independent dimerization was demonstrated for other RTKs as well [3,45,46,47]. It appears that the spatial distribution of RTKs, either dictated by ligands or by other factors, regulates the signal propagation and cell fate. 

The ligand-induced FGFRs’ dimerization induces conformational changes within the intracellular regions of the receptor that trigger sequential tyrosine autophosphorylation events. The initial modification of tyrosine 653 largely increases the activity of the receptor kinase, which results in the phosphorylation of other tyrosine residues—Y583, Y463, Y766, Y585, and Y654 [48]. This set of modifications further increases the enzymatic activity of the kinase domain, leading to the phosphorylation of Y677 and Y766 [48]. The phosphorylated tyrosine residues serve as docking sites for the downstream signaling proteins—phospholipase C-gamma (PLCγ) and the signal transducer and activator of transcription (STAT) [21,22,49,50,51], whereas the adaptor protein FRS2 binds to the juxtamembrane domain of FGFRs [52]. 

### 2.3. FGFRs-Dependent Signaling Pathways

The propagation of signals by the activated FGFRs occurs through the receptor tyrosine kinase-mediated phosphorylation of the adaptor proteins of the pivotal cellular signaling pathways, namely: PLCγ, STAT, phosphoinositide 3-kinase (PI3K)/protein kinase B (AKT)/mammalian target of rapamycin (mTOR), and Ras–Raf–MEK–ERK (Figure 1). FGFR-phoshorylated PLCγ hydrolyses phosphatidylinositol 4,5-bisphosphate (PIP_2_), generating the following secondary messengers: inositol triphosphate (IP_3_) and diacylglycerol (DAG). DAG activates protein kinase C (PKC), whereas IP_3_ alters the cellular calcium levels. The PLCγ signaling modulates a plethora of cellular processes, including cell division, calcium homeostasis, remodeling of cell membranes, and cytoskeleton [53,54,55]. The FGFRs activate the STAT signaling pathway by phosphorylating STAT1, 3, and 5 [56]. STAT signaling modulates diverse cellular functions, like cell survival and proliferation [57]. The phosphorylation of FRS2 by FGFRs stimulates the binding of the growth factor receptor bound-2 (GRB2) protein to FRS2. GRB2 propagates signals through the following two major signaling pathways: PI3K/AKT/mTOR and Ras–Raf–MEK–ERK. The recruitment of GAB1 to GRB2 initiates the PI3K/AKT/mTOR cascade that regulates cell division, differentiation, apoptosis, and autophagy, and is one of the most frequently altered signaling pathways in cancer [58,59]. GRB2 binding by son of sevenless (SOS) activates Ras–Raf–MEK–ERK pathway, which modulates cell proliferation, migration, differentiation, and survival [60].

The FGFRs-dependent signals activate several transcription factors, like c-myc, c-jun, and c-fos [22]. The list of downstream genes whose expression is modulated by FGFs–FGFRs is still expanding, and includes, among the others, signaling proteins, growth factors and their receptors (also FGFs–FGFRs), and transcription factors [61,62,63,64].

The cellular response elicited by FGFRs depends on the FGFR and FGF type that together assemble the signaling complex [21,22]. Recently, Zinkle et al. proposed a “threshold” model, where the stability of the ligand-induced FGFR dimer dictates the cellular outcome triggered by the activated signaling cascades [4]. Short-lived FGFRs dimers (with transient signaling) are capable of promoting an anti-apoptotic response, whereas cell division and migration require stable dimer generating prolonged signaling [4]. The strength of the FGFRs signaling is further modulated at the level of protein synthesis, trafficking, and degradation, as well as by co-receptors and partner proteins [37].

## 3. Dysregulation of FGFRs in Cancers

The disrupted homeostasis of the FGFRs signaling system results in uncontrolled cell behavior. Aberrant FGFRs function was detected in a large number of various cancer types, including breast, lung, ovarian, head and neck, and prostate tumors [65]. The dysregulation of FGFRs was detected in over 7% of cancers in recent large-scale high-throughput studies [66]. The imbalanced FGF/FGFRs signaling may be of a ligand-independent nature (e.g., enhanced signal transmission by overproduced or mutated FGFRs). or may be caused by dysregulated ligands.

### 3.1. Overexpression of FGFRs 

**Figure 2:**
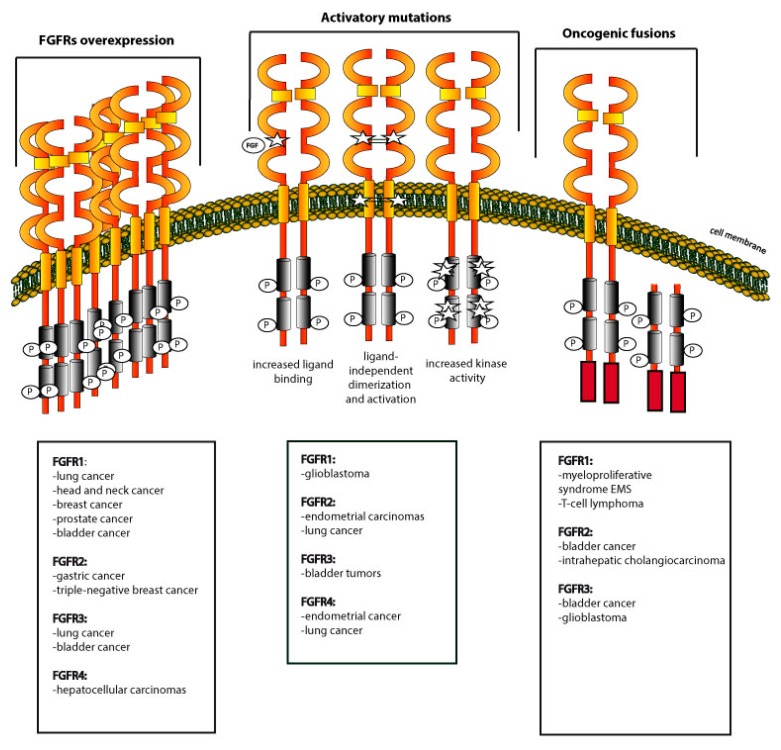
Dysregulation of FGFRs in cancer. FGFRs overexpression, mutations, and fusion proteins are often identified in various tumors, and lead to uncontrolled, oncogenic signaling. The most frequently found FGFRs alternations in particular cancers are indicated below the scheme.

The increased level of FGFRs on the cancer cell surface may facilitate in capturing the circulating FGFs, thus leading to a hyperactivation of signaling. Alternatively, overproduced FGFRs can cause FGFRs autoactivation in a ligand-independent manner (Figure 2). The overexpression of FGFRs has been detected in a plethora of human tumor types, like lung, brain, head and neck, prostate, and breast cancers [67]. 

The amplification of *FGFR1*, resulting in receptor overproduction, was found in small-cell lung carcinoma and squamous non-small cell lung carcinoma (about 5% and 20% of reported cases, respectively) [68,69]. The overexpression of FGFR1 was detected in over 80% of human papilloma virus (HPV)-positive and in 75% of HPV-negative head and neck squamous cell carcinoma [70,71]. The increased level of FGFR1 is observed in up to 15% of human breast cancers, especially in more invasive breast cancers, and may be one of the oncogenic drivers [72,73,74,75,76]. The overexpression of FGFR1 was also frequently observed in prostate cancers, bladder cancers, and, rarely, in osteosarcomas [67,77,78,79,80].

The overproduction of other FGFR members is less frequent. The overexpression of FGFR2 was detected in up to 10% of gastric cancers and in 4% of triple-negative breast cancers [81,82]. The elevated level of FGFR2 in invasive gastric tumors facilitates the constitutive activation of this receptor, and is associated with a poor patient prognosis [83]. FGFR2 is also overexpressed by the majority of melanoma cell lines [84]. FGFR3 is overexpressed in about 50% of oral squamous cell carcinoma, in the early stage of non-small cell lung cancers, and in bladder tumors [70,71,85,86]. The overexpression of FGFR4 was identified in over 50% of hepatocellular carcinoma, where it contributes to the invasiveness of cancer cells [87,88,89,90]. FGFR4 levels are largely increased in various melanoma cell lines [84].

### 3.2. Activating Mutations within FGFRs

Up till now, numerous mutations in genes encoding FGFRs have been detected [91,92,93,94]. Interestingly, mutations occur in various regions of the FGFRs and differentially affect receptor function. Typically, mutations in the FGFRs cause receptor over-activation either by facilitating FGFRs interaction with FGFs (mutations in the extracellular region of the receptor), or by causing constitutive receptor activation (Figure 2). Activating mutations in *FGFR1* were found in glioblastoma [95,96], *FGFR2* is often mutated in endometrial carcinomas and lung cancers [97,98], *FGFR3* mutations are frequently found in bladder tumors and melanoma [99,100], whereas *FGFR4* is mutated in endometrial and lung cancers [101,102]. The mechanisms behind the carcinogenic activity of the representative FGFRs mutants are described below.

One of the best characterized FGFRs mutations is the S252W substitution within FGFR2. This somatic mutation is frequently identified in patients with endometrial carcinomas, and is identical to the germline mutation causing Apert Syndrome [97]. Serine 252 is located in the flexible linker connecting the D2 and D3 domains of FGFR2, and participates in ligand binding. The S252W mutation enhances the affinity and alters the selectivity of FGFR2 towards FGFs (Figure 2) [103,104]. Moreover, this single point mutation facilitates autocrine FGFR2 signaling by allowing for the recognition of mesenchymal ligands by mesenchymal FGFR2, and the binding of epithelial ligands to epithelial FGFR2 [104]. 

The S249C and R248C variants of FGFR3 are often found in bladder cancers [105]. These mutations introduce additional cysteine residues in the extracellular region of FGFR3 that cause the formation of a disulfide bridge between two FGFR3 monomers. The FGFR3 S249C dimer is activated in a ligand-independent manner that leads to a transformation of fibroblasts into tumor cells (Figure 2) [106]. 

Amino acid substitution in the transmembrane region of FGFR4 (Y367C) was identified in breast cancer cells. FGFR4 Y367C forms ligand-independent dimers characterized by the constitutive activation of the receptor and downstream signaling pathways [107]. The introduction of unpaired cysteine residues in the transmembrane domain of FGFR3 also leads to ligand-independent uncontrolled signaling in different tumors (Figure 2) [108]. 

The intracellular region of FGFRs, especially the activation loop split tyrosine kinase domain, is frequently mutated in various cancer types, including glioblastoma, endometrial carcinoma, lung, and bladder cancer [94]. Mutations in the activation loop of FGFRs, K655I and K656D/M/N/E in FGFR1, K650E/M/N in FGFR2, and K652E/M/N/Q/T in FGFR3, largely increase the receptor kinase activity and downstream signaling (Figure 2) [94,108,109]. 

Not only gain-of-function mutations in the FGFRs are found in cancers. The loss-of-function mutation A649T in the FGFR2 kinase domain was identified in melanoma. This mutation blocks the receptor autoactivation and signaling, but its relevance for the cell transformation is unknown [110]. 

### 3.3. Oncogenic Fusions of FGFRs

The chromosomal translocations may result in the expression of FGFRs fusion proteins that, depending on the fusion partner, display a different subcellular localization and function. Up till now, numerous FGFR fusions with diverse partner proteins have been reported in various cancer types, and account for 8% of the alterations within FGFRs [66,94]. The chromosomal translocations t(8;13)(p11;q12), t(8;9)(p11;q33), t(6;8)(q27;p11), and t(12;8)(p11;p11p22)) observed in highly invasive 8p11 myeloproliferative syndrome (EMS), fuse ZNF198, CEP110, FOP1, and FOP2 to the tyrosine kinase domain of FGFR1 [111,112,113]. In addition, FGFR1–FOP1 and FGFR1–FOP2 are found in lung cancer and leukemia [114,115]. An oncogenic FGFR2–PPHLN1 fusion was detected in 16% of intrahepatic cholangiocarcinoma, a bile duct cancer with poor patient prognosis [116]. FGFRs fusions with TACC proteins were found in bladder cancer and in glioblastoma multiforme [117,118]. Moreover, FGFRs fusion proteins were detected in metastatic breast, lung, and prostate cancer patients [119].

Typically, the partner protein within the FGFRs fusions contains an oligomerization domain, causing receptor autophosphorylation and the initiation of downstream signaling cascades (Figure 2). The most common oligomerization domains found in FGFR fusion proteins are coiled-coiled motifs, zinc finger motifs, and leucine zippers that position FGFRs kinase domains in an appropriate orientation for kinase activation [94]. Usually, FGFRs fusions activate signaling pathways that are under the control of typical FGF/FGFR signaling. However, becaause the structural alterations in the FGFRs within FGFRs fusions, the activation of a particular pathway may be blocked or the intensity of a signal propagation through certain pathways may differ from classical FGFRs signaling. For example, the FGFR3 fusions with transforming acidic coiled–coil containing 3 (TACC3) are devoid of C-terminal tyrosine, which is critical for the interaction with PLCγ. Thus, the FGFR3–TACC3 fusion was incapable of activating the PLCγ pathway in bladder cancer cells [118]. A number of FGFR1 fusion proteins display altered signaling, as they are devoid of the FRS2 binding site [120]. The FGFRs fusions differentially activate the isoforms of STAT proteins [121,122]. Besides changing the specificity of the signaling pathways, the incorporation of a fusion partner to FGFRs may alter the subcellular localization of FGFRs. While FGFRs are typically plasma membrane proteins, ZNF198–FGFR1, FOP1–FGFR1, FOP2–FGFR1, and TEL-FGFR3 are cytoplasmic proteins because of the removal of the FGFR1 transmembrane domain [111,112,113,114,115,123]. In the FGFR3–TACC3 fusion, the TACC3 component dimerizes the receptor kinase and targets FGFR3–TACC3 to the mitotic spindle pole or nucleus [11,124]. 

## 4. Cellular Trafficking of FGFRs

The specificity of the FGFRs’ function depends on the subcellular localization of these proteins. Recent reports imply that FGFRs are present in various subcellular organelles, where they facilitate cancer development [125,126]. At steady state conditions, a majority of FGFRs molecules are present on the cell surface. FGFRs can be internalized via diverse endocytic events that result in the generation of an endosomal pool of receptor. These molecules can be subsequently degraded or recycled to the plasma membrane. Besides the endomembrane system, FGFRs are found in the nucleus and in the mitochondria, where these receptors contribute to carcinogenic processes [126,127,128]. In this section, we summarize the current state of knowledge about FGFRs trafficking.

### 4.1. Internalization of FGFRs

The endomembrane secretion system directs the vast majority of RTK molecules (including FGFRs) to the plasma membrane. This is attributed to the presence of cleavable N-terminal signal sequences within the RTKs that target receptors to the secretory route, as well as because of the transmembrane domain that embeds RTKs in the cell membranes. RTKs undergo constitutive endocytosis (internalization) from the plasma membrane, which occurs at low, basal rates. The rate of constitutive RTKs endocytosis is lower than their synthesis and recycling, which decides about the accumulation of receptors on the plasma membrane [129].

**Figure 3:**
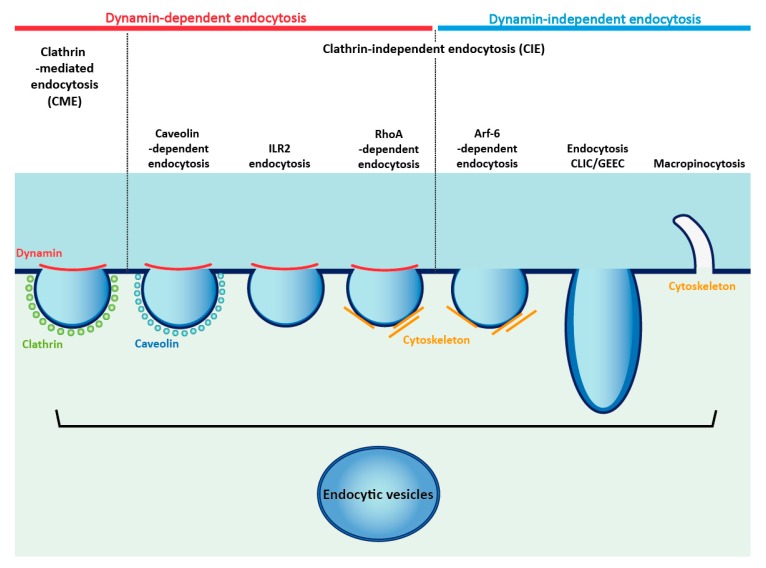
Cellular internalization pathways utilized by RTKs. Endocytic pathways may involve dynamin, or may be independent of this protein. Various mechanisms lead to the formation of intracellular endocytic vesicles containing RTKs (for details, see main text of the manuscript).

Ligand binding largely accelerates the RTKs internalization, which may occur via several endocytic pathways that differ depending on the molecular mechanism and scale of the endocytic event (Figure 3) [129,130,131,132]. The cellular internalization routes can be divided into two major groups depending on the involvement of the GTPase-dynamin [133]. The vast majority of the plasma membrane receptors and their ligands utilize dynamin-dependent, clathrin-mediated (CME), or caveolin-mediated endocytosis for their internalization [130,134]. The interleukin-2 receptor (IL2R) is internalized via a unique pathway that requires dynamin, but does not involve clathrin or caveolin [135]. The endocytic pathway utilizing dynamin and Arf-6 is responsible for the internalization of a number of plasma membrane-resident proteins, like CD44, CD55, CD98, CD147, Glut1, and ICAM1 [136]. Large volumes of extracellular fluid containing diverse macromolecules are internalized by micropinocytosis, which is independent of dynamin, clathrin, and caveolin, but requires the reorganization of cytoskeleton [137]. The plasma membrane proteins anchored by glycosylphosphatidylinositol (GPI), CD44, and integrins are internalized by an endocytic pathway utilizing clathrin independent carriers CLIC [132]. The proper functioning of the endocytic processes is crucial for cellular homeostasis, and is implicated in cancer development, for example, because of the dysregulated RTKs signaling [138,139,140].

**Figure 4:**
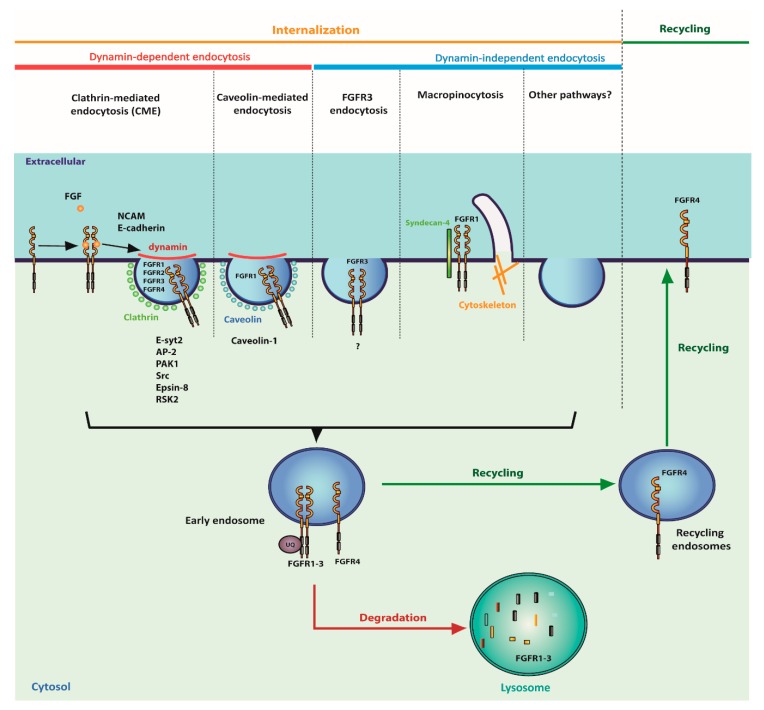
Internalization and cellular trafficking of FGFRs. FGFRs are internalized via diverse endocytic mechanisms. The selection of an internalization pathway depends on the receptor type and is modulated by the partner proteins. After internalization, FGFRs are sorted into endosomes, where they can be directed either to lysosomal degradation (mainly FGFR1-3) or recycled to the plasma membrane (FGFR4). The receptor ubiquitination decides about the intracellular sorting of endocytosed FGFRs.

FGFRs undergo constitutive internalization from the plasma membrane. However, the binding of the FGFs and the subsequent receptor activation stimulates FGFRs endocytosis [141,142,143,144]. Depending on the ligand and FGFR isoform, different endocytic pathways are engaged in FGFRs internalization (Figure 4). However, FGFRs mainly utilize CME as a way to reach the cellular interior [142,144]. 

The extended synaptotagmin-2 (Esyt2) was identified as a binding partner of FGFR1 and a major regulator of FGFRs CME. Esyt2 interacts with components of the adaptin-2 complex (AP-2), which constitutes the central adaptor for CME. The ligand-induced rapid internalization of FGFRs is dependent on Esyt2 [145]. The interaction between FGFR1 and other E-Syt isoforms was demonstrated as well [146]. Interestingly, E-Syt2 interacts with p21-GTPase activated kinase (PAK1), and recruits PAK1 to specific phospholipid domains at the plasma membrane. PAK1 is known for its function in the remodeling of actin cytoskeleton, which implicates the possible involvement of Esyt2 and PAK1 in the cytoskeleton remodeling-dependent internalization of FGFRs [147]. The CME of FGFR1 and FGFR2 is modulated by non-receptor type kinase Src. The ligand-induced activation of FGFR1 stimulates the recruitment of Src to FGFR1–FRS2 complexes. Upon internalization, FGFR1 is released from the complexes containing Src. The Src-mediated FGFR1 internalization requires the presence of an intact actin cytoskeleton [148]. Following fibroblast growth factor stimulation, the number of clathrin-coated pits (CCPs) increases, and FGFR2 is rapidly sequestered into assembled CCPs. Subsequently, FGFR2 is sorted into endosomes via CME. FGFR2 internalization via CME, similarly to FGFR1, requires Src, but also involves epsin-8 (Figure 4) [144].

Although CME seems to play a fundamental role in FGFRs endocytosis, other endocytic routes were implicated in the internalization of these receptors as well (Figure 4). FGFR1 was detected in caveolae, where it interacts with caveolin-1. FGF2 binding releases FGFR1 from the complex with caveolin-1, and this step is important for the spatiotemporal regulation of FGFR1-dependent signaling [149]. *Rickettsia rickettsia*, pathogenic Gram-nagative bacteria causing spotted fever in humans, interact with FGFR1 on the surface of the host cells. It is noteworthy that the bacteria are internalized via the FGFR1-dependent, caveolin-mediated endocytic pathway [150]. Syndecan-4 (S4) is a heparan sulfate proteoglycan involved in cell migration and adhesion. In the presence of FGF2, S4 forms a complex with FGFR1, which promotes the internalization of the FGF2–FGFR1–S4 by macropinocytosis, affecting the FGFR1 levels and downstream signaling [151]. 

Interestingly, an FGFR1 cellular uptake independent of clathrin, caveolin, and macropinocytosis was observed as well, indicating the possible engagement of other endocytic pathways [152]. The kinetics of FGFR3 endocytosis is slower in comparison with the internalization of FGFR1, and involves both CME and clathrin-independent endocytosis (CIE). However, the CIE route for FGFR3 internalization was not identified. It is noteworthy that this pathway is independent of several known CIE proteins—Arf6, flotillins, and Cdc42 [153]. 

The signals within the FGFRs that trigger endocytosis may also differ, depending on the receptor type. It was demonstrated that the inhibition of the FGFRs receptor tyrosine kinase only partially decreased the internalization of FGFR1 and FGFR4, while it largely inhibited the uptake of FGFR2 [141,144,154]. We have recently demonstrated that the dimerization of FGFR1, but not the activation, constitutes the signal for the CME of this receptor. Bivalent engineered anti-FGFR1 antibodies that cause receptor dimerization without concomitant autophosphorylation were efficiently internalized via CME. The efficiency of the CME of the antibody-FGFR1 complexes was not altered by the inhibition of the FGFR kinase with small-molecule chemical inhibitor, or by the kinase-dead mutation within FGFR1 [13]. In agreement with our findings, Esyt2 recognizes the special conformation of FGFRs achieved upon receptor activation, however, the receptor autophosphorylation is not necessary for the Esyt2-FGFRs interaction [145,146]. 

The internalization of FGFRs may be tailored by interaction partners. A neural cell adhesion molecule (NCAM) interacts with FGFR1, influencing the receptor internalization. The decreased FGFR1 endocytosis caused by NCAM binding leads to a sustained receptor-dependent signaling and cell migration [155]. In epithelial cells, FGFR1 is internalized together with E-cadherin, and this co-endocytosis is relevant for the efficiency of FGFR1 internalization and receptor function [156]. The internalization of FGFR1 is also modulated by cellular signaling. It was demonstrated that the activated p90 ribosomal S6 kinase 2 (RSK2) directly interacts with the intracellular domain of FGFR1. RSK2 phosphorylates FGFR1 on serine 789, which facilitates the receptor endocytosis [157]. 

The cellular fate of the internalized FGFRs largely depends on the receptor type and bound ligand. FGF1–FGFR1 displays the fastest trafficking to lysosomes, where the degradation of these proteins takes place. FGF1 in complex with FGFR2 or FGFR3 is also directed to the degradative pathway, but the kinetics of their lysosomal sorting is lower in relation to the FGF1–FGFR1 pair. In contrast, the endocytosed FGF1–FGFR4 complexes are sequestered into recycling endosomes. Interestingly, FGFR4 lacks several lysine residues present in FGFR1–FGFR3, and is less efficiently ubiquitinated, which may stand for the decreased lysosome targeting of this receptor [158]. In agreement with this hypothesis, the ubiquitination-deficient mutants of FGFR1 were endocytosed at normal rates, however, their sorting into lysosomes was hampered with the concomitant increased receptor recycling [159]. Hrs (hepatocyte growth factor-regulated tyrosine kinase substrate), a component of the endosomal sorting complex required for transport-0 (ESCRT-0), which recognizes ubiquitinated proteins, was implicated in the FGFRs intracellular sorting [160]. The fate of the internalized FGFRs also depends on the growth factor that induces receptor endocytosis. The FGF7–FGFR2b complex is directed to the lysosomes for degradation, whereas the FGF10–FGFR2b complex is recycled to the plasma membrane [142].

Despite the large progress regarding FGFRs biology, the knowledge about FGFRs internalization is still far from complete. Recent high throughput proteomic screens for FGFRs interaction partners revealed novel proteins that may regulate FGFRs trafficking [161,162]. Still, further analyses are required for understanding the molecular mechanisms behind FGFRs internalization and sorting. 

### 4.2. Transport of FGFRs to the Nucleus

Although RTK were considered for years as bona fide plasma membrane proteins, a large number of reports provided evidence that these receptors can be localized inside the cells, mainly in the nucleus. The representatives of over 12 RTK subfamilies have been found in the nucleus, where they fulfil diverse functions, and are also related to cancer development and chemotherapy resistance [163]. 

It is noteworthy that there are several mechanisms utilized by RTKs to reach the nucleus. Nuclear RTK fragments can be generated via alternative splicing, or the proteolytic cleavage of plasma membrane-localized full-length receptors with caspases, secretases, granzymes, and other proteases [164]. Full length RTKs can reach the nucleus as well, either via vesicular pathways or after retrotranslocation from the ER to the cytosol [163,165]. Inside the nucleus, RTKs act either as kinases, phosphorylating a number of regulatory proteins, or function as transcription regulators, affecting cell proliferation, apoptosis, and migration in a kinase-independent manner [163,165]. Importantly, the nuclear localization of RTKs in various cancers is associated with poor prognosis [166,167,168]. 

**Figure 5:**
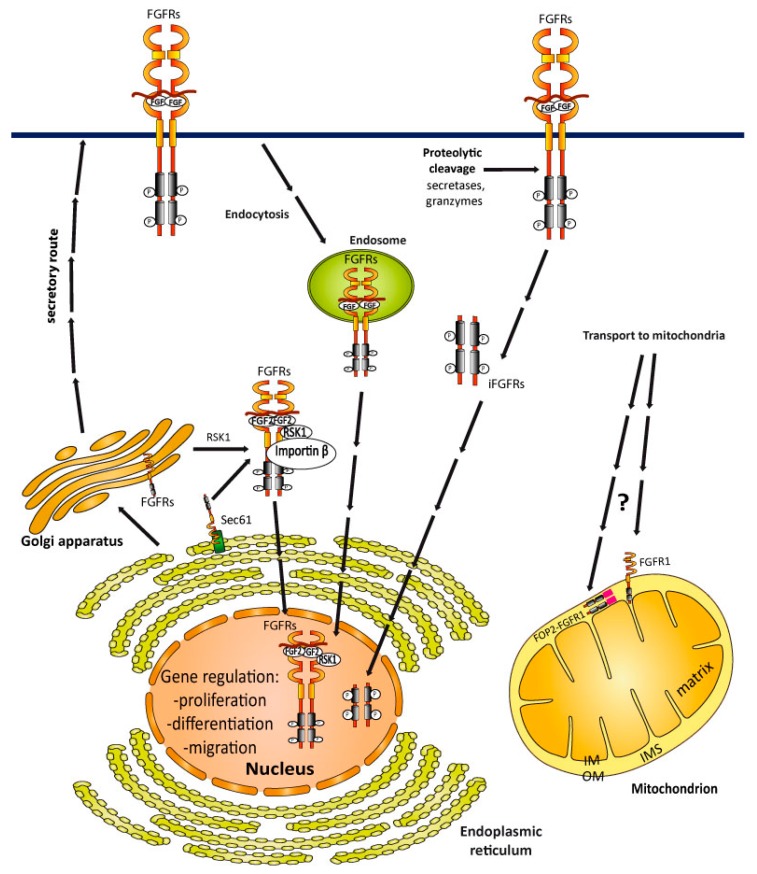
Intracellular transport of FGFRs. FGFRs are synthesized at the ER, where FGFRs enter a secretory route that directs them to the plasma membrane. At the ER/Golgi, FGFRs can retrotranslocate to the cytosol with the help of RSK1 and FGF2, and can subsequently enter the nucleus to directly regulate gene expression. Full length FGFRs can reach the nucleus after endocytosis. Alternatively, proteolytic processing releases soluble fragments of FGFRs that are targeted to the nucleus. Full length FGFR1 is partially localized to the mitochondria in cancer cells. The molecular mechanism utilized by FGFR1 to integrate into the mitochondrial outer membrane (OM) is unknown. The FOP2–FGFR1 fusion protein is also targeted to the mitochondria, but in contrast to the full length FGFR1, FOP2–FGFR1 is localized to the mitochondrial intermembrane space (IMS). Up till now, FGFRs have not been found in the mitochondrial matrix and in the inner membrane (IM).

FGFRs were detected in the nuclei of a number of different cell types [125,127,128,169,170,171,172]. It is noteworthy that in most cases, full length FGFRs enter the nucleus [125,173]. The molecular mechanisms utilized by particular FGFRs for their nuclear transport are unclear, and conflicting data point to the different trafficking possibilities for FGFRs (Figure 5). One of possible mechanisms employed by the full length FGFRs in nuclear translocation involves the retrotranslocation of FGFRs from the ER/Golgi compartments [125]. Typically, after co-translational insertion into the ER membranes, FGFR1 traffics via the vesicular transport systems through the Golgi apparatus, and finally reaches the plasma membrane. This process may be accompanied by the retrotranslocation of the pool of FGFR1 into the cytosol. At the ER/Golgi, FGFR1 may utilize a retrograde transport system through the Sec61 channel, similarly to ER-associated protein degradation (ERAD) [125,174]. Once in the cytosol, FGFRs interact with ribosomal S6-kinase 1 (RSK1) and FGF2, which facilitate receptor transport to the nucleus (Figure 5) [127,128,175,176]. The nuclear translocation of FGFRs involves importin β [171].

Alternatively, full length FGFRs may be transported to the nucleus from the cell surface. The internalization of FGFRs via CME or CIE may facilitate the nuclear localization of FGFRs [156,177]. The exact mechanism explaining how the endosomal pool of FGFRs is translocated to the nucleus in not clear, however, retrograde vesicular transport resembling viral entry can be used (Figure 5) [178]. 

The third pathway utilized by FGFRs to enter the nucleus is based on the generation of intracellular receptor fragments. It was demonstrated that the stimulation of FGFR3 with FGF1 induces sequential receptor proteolysis. First, the FGFR3 extracellular domain is cleaved off by an unknown protease, then intramembrane proteolysis by γ-secretase releases the C-terminal, soluble receptor fragment to the cytosol. Next, the FGFR3 intracellular fragment is transported to the nucleus (Figure 5) [179]. A similar mechanism was observed for FGFR1 in breast cancer cells [180].

The function of nuclear FGFRs largely depends on the cell type. In the neurons, nuclear FGFR1 regulates the gene expression that governs cell proliferation and differentiation [125]. Nuclear FGFRs are also implicated in cancer development. Pancreatic stellate cells (PSCs) form dense stroma that surround pancreatic cancer cells, limiting tumor response to chemotherapy. FGFR1 and FGF2 localize to the nucleus in the PSCs cells, and the nuclear localization of FGFR1/FGF2 is critical for the invasive properties of PSCs [127,128]. The proteolytic cleavage of FGFR1 by granzyme-B yields the receptor fragment that is targeted to the nucleus in invading breast tumors. Nuclear FGFR1 regulates the transcription of a precise set of target genes critical for aggressive behavior of cancer cells [180]. 

### 4.3. FGFRs Sorting into Mitochondria

Mitochondria are organelles that perform numerous functions vital for cell homeostasis, like energy conversion, calcium ions regulation, biogenesis of iron-sulfur clusters, metabolism, and programmed cell death. This versatility of mitochondrial functions may be achieved by the dynamic properties of the mitochondrial proteome [181]. Mitochondria are also integrated into cellular signaling systems [182]. Importantly, multiple RTKs can be transported to the mitochondria, adjusting the cell metabolism and apoptosis [183]. The understanding how RTKs travel to these organelles is still scarce. 

Importantly, a fraction of FGFR1 were identified in the mitochondria of lung cancer cells (Figure 5). The biochemical experiments revealed that FGFR1 is mainly localized to the mitochondrial outer membrane (OM). Besides full length FGFR1, an oncogenic FOP2–FGFR1 fusion was also found in the mitochondria of lung tumor cells. However, FOP2–FGFR1 was present mainly in the mitochondrial intermembrane space (IMS). How these proteins reach the mitochondria is unknown [126]. Importantly, mitochondrial FGFR1 promotes the growth of cancer cells by adjusting the energy metabolism. Many tumors generate energy via glycolysis instead of using mitochondrial oxidative phosphorylation (so called Warburg effect). At the mitochondria, FGFR1 binds to and phosphorylates pyruvate dehydrogenase kinase 1 (PDHK1) at multiple tyrosine residues. This modification enhances the activity of PDHK1, leading to the de-activation of pyruvate dehydrogenase complex (PDC), thus switching off the mitochondrial oxidative phosphorylation and promoting the Warburg effect [126]. Notably, the mitochondrial localization and function of FGFR1 is independent of growth factor stimulation. It is likely that, besides PDHK1, FGFR1 also phosphorylates other mitochondrial proteins. The presence of the constantly active FOP2–FGFR1 fusion in the mitochondria further supports a more complex function for FGFRs in the mitochondria. 

## 5. Therapeutic Strategies against Cancers with Abnormal FGFRs

As altered FGFs/FGFRs signaling is frequently observed in various tumors, FGFRs became attractive molecular targets for the development of diverse anti-cancer therapeutics, excellently reviewed in the literature [184]. Typically, anti-FGFRs therapeutic strategies aim at the inhibition of ligand-dependent FGFRs activation, either by interfering with FGFs–FGFRs interaction or by blocking receptor kinase activity. These effects are usually achieved by using small-molecule chemical tyrosine kinase inhibitors (TKIs), FGF ligand traps, therapeutic antibodies, aptamers, or antagonistic peptide mimics [185]. 

TKIs are by far the most intensively explored anti-FGFRs agents, because of their simplicity and low costs of production [67]. Unfortunately, the clinical and preclinical trials indicate that cancer cells develop diverse resistance mechanisms against TKIs [186,187]. The activation of downstream signaling cascades in the presence of anti-FGFRs TKIs, so called bypass signaling, is achieved by the overexpression of other RTK members or by mutations within the TKIs binding pocket in FGFRs. Alternatively, heterogeneity in FGFRs expression within the tumor may facilitate the development of resistance to TKIs [184]. Moreover, the FGFRs present inside the cells may promote cancer growth independently of tyrosine kinase activity. Thus, novel anti-FGFRs anticancer strategies that, instead of blocking FGFRs’ kinase activity, directly lead to cancer cell death, constitute highly promising approaches. 

### 5.1. Employing Internalization of FGFRs for Selective Treatment of FGFR-Dependent Cancers

The elevated level of FGFRs on the surface of the cancer cells allows for the selective recognition of tumor entities by engineered molecules targeting these receptors. Interestingly, the naturally occurring cellular trafficking of FGFRs may serve as a highly efficient mechanism for the downregulation of receptor levels on the cell surface, and for the delivery of cytotoxic drugs into cancer cells. In this section, we focused on hijacking the cellular trafficking of FGFRs for the selective treatment of tumors with aberrant FGFRs. 

**Figure 6:**
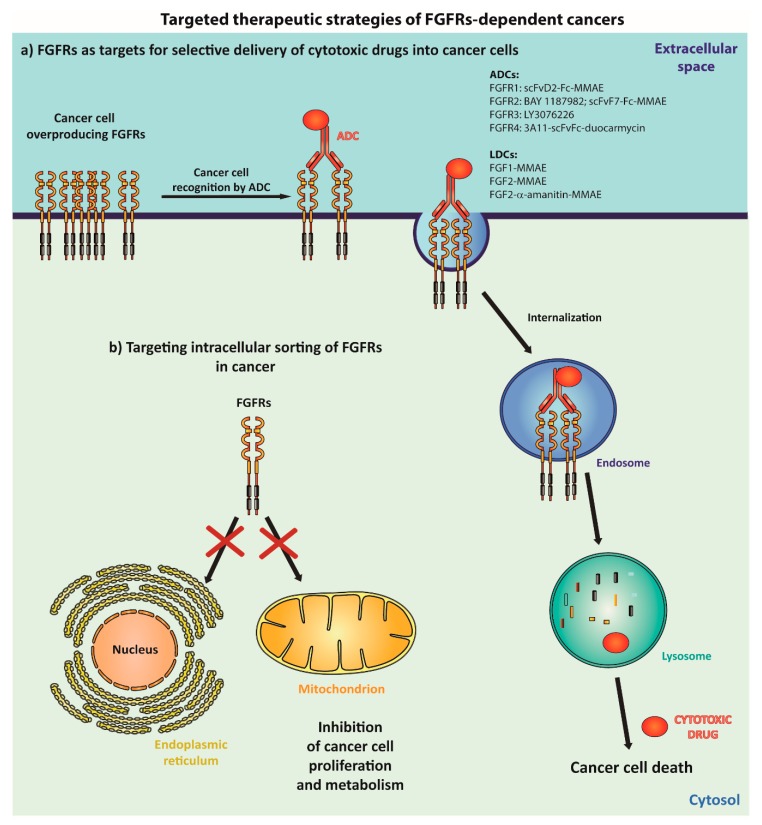
Employing cellular protein trafficking machineries for targeted therapeutic strategies of FGFRs-dependent cancers. Numerous cancers overproduce FGFRs on their surface. Construction of highly selective cytotoxic molecules, like antibody-drug conjugates (ADCs) or ligand drug conjugates (LDCs) allows for the specific recognition of cancer cells and for the delivery of cytotoxic agents into the cancer cell interior, resulting in cell death (**a**). FGFRs are found in the nucleus and mitochondria, where they contribute to oncogenic process. The inhibition of FGFRs transported to these organelles can block the oncogenic activity of intracellular FGFRs (**b**).

Antibody-drug conjugates (ADCs) constitute a novel group of anticancer therapeutics. ADC is composed of antibody and cytotoxic drugs connected by a specifically designed linker [188,189]. Antibodies allow for the selective recognition of cancer cells overproducing antigens, and facilitate in the cellular uptake of ADCs via receptor-mediated endocytosis. ADCs are then targeted to lysosomes for degradation, and the subsequent release of drug moieties occurs. Cytotoxic drugs escape from the lysosomal lumen, reaching their intracellular target and causing cancer cell death (Figure 6). 

Up till now, several ADCs targeting FGFRs have been constructed, and their cytotoxic potential was evaluated (Figure 6). FGFR1-specific antibodies have been obtained using a phage display approach with Tomlinson libraries [190]. The most promising antibody, bivalent scFvD2-Fc, binds with the sub-nanomolar affinity to the epitope localized within the N-terminal region of the D1 domain of FGFR1 [191,192]. Interestingly, scFvD2-Fc induces FGFR1 dimerization without receptor activation, which results in rapid FGFR1 internalization mainly via CME [13]. Antibody-induced CME of FGFR1 targets the scFvD2-Fc-FGFR1 complexes to lysosomes, leading to a downregulation of the FGFR1 levels on the cell surface [13,190]. Thus, the FGFR1-specific scFvD2-Fc antibody alone has a therapeutic potential, as it is able to decrease the FGFR1 levels on the cancer cell surface by inducing the cellular trafficking-dependent lysosomal degradation of the receptor. scFvD2-Fc was used as a targeting molecule in the ADC approach, with monomethyl auristatin E (MMAE) as a cytotoxic agent. scFvD2-Fc-MMAE displays cytotoxic activity against lung cancer cells with FGFR1 overproduction [190]. 

The FGFR2-specific antibody, BAY 1179470, was selected via the phage display approach using the n-CoDeR Fab library [193]. BAY 1179470 recognizes with a high affinity the extreme N-terminus of FGFR2. BAY 1179470 interacts with FGFR2 on the surface of the cancer cells overproducing this receptor and induces FGFR2 internalization and the subsequent lysosomal degradation. BAY 1179470 was used as a targeting molecule for the generation of ADC containing *N*-methyl auristatin-W as a cytotoxic payload (BAY 1187982). BAY 1187982 demonstrates a high cytotoxicity against cancer cells producing FGFR2, and the cytotoxic activity correlates well with the level of FGFR2 on the cell surface, underscoring the relevance of receptor trafficking. FGFR2 is overproduced in gastric cancers and in triple negative breast cancers. BAY 1187982 displays a selective cytotoxic potential in gastric and triple negative breast cancer xenograft models, and in mouse cancer models [193]. Another ADC targeting FGFR2 was recently developed. A FGFR2-specific scFvF7-Fc bivalent antibody was obtained by phage display using Tomlinson libraries [194]. scFvF7-Fc recognizes the epitope within the D1 domain of the receptor with sub-nanomolar affinity. scFvF7-Fc binds FGFR2 on the cell surface and is taken up by the cells in a receptor-dependent manner. scFvF7-Fc-MMAE ADC was constructed. This conjugate displays a selective anticancer activity in the in vitro experiments [194]. LY3076226 is a novel ADC developed by Lilly Oncology Company, which is composed of a human monoclonal antibody against FGFR3 conjugated to a microtubule inhibitor—DM4. Currently, LY3076226 is in phase I clinical trials against advanced and metastatic cancers (NCT02529553). The construction of ADC composed of a chimeric antibody, 3A11 scFv-Fc, and duocarmycin for targeting of FGFR4 in rhabdomyosarcom was recently reported [195]. 

Not only antibodies or their engineered fragments have been used as targeting molecules for the selective delivery of cytotoxic drugs into cancer cells overproducing FGFRs. FGF1 and FGF2, natural FGFRs ligands, were successfully conjugated with MMAE in a site-specific manner, forming ligand drug conjugates (LDCs). The FGF-based conjugates retained the receptor binding and cellular trafficking properties of unconjugated FGFs, and demonstrated a selective cytotoxic potential against FGFR1-overproducing lung cancer cell lines [196,197,198,199]. Anti-FGFR1 aptamers were also developed for the specific recognition of FGFR1-overproducing cancers. When fused to the supramagnetic conjugates, anti-FGFR1 aptamers displayed a selective cytotoxic potential against the model cells overproducing FGFR1 [200].

### 5.2. Targeting the Intracellular Sorting of FGFRs into Nucleus and Mitochondria in Cancer

Besides the cell surface, FGFRs are localized in the nucleus and in the mitochondria, where they promote oncogenesis by directly regulating the gene expression or by adjusting the cellular metabolism (Figure 5). Importantly, the intracellular functions of FGFRs in cancer may be independent of the receptor kinase activity, limiting the applicability of most of the frequently used anti-cancer agents, like TKIs. Thus, the specific inhibition of FGFRs’ translocation into the nucleus and mitochondria may constitute a promising therapeutic strategy against various tumors (Figure 6). One of the possible routes employed by FGFRs to reach the nucleus is the generation of receptor intracellular fragments by processing proteases of the plasma membrane. The inhibition of processing the proteases was implicated in the treatment of cancers with nuclear RTKs [164].

Breast cancer cells contain FGFR1 truncation, which resides in the nucleus. The nuclear FGFR1 arises from the proteolytic processing of the full-length receptor by granzyme B. Importantly, the synthetic peptide inhibitor specific towards granzyme B blocked FGFR1 processing, nuclear translocation, and the migration of cancer cells [180]. Components of the nuclear protein transport systems, including importins, are targets of anticancer therapies [201]. Nuclear FGFRs utilize importin-β for their translocation [171]. Targeting importin-β reduced the nuclear migration of FGFR1 and resulted in the inhibition of cell proliferation, highlighting the therapeutic potential of this approach [171]. 

In the mitochondria of cancer cells, FGFR1 promotes the Warburg effect by phosphorylating PDHK1 [126]. Besides FGFR1, the FOP2–FGFR1 fusion protein is also found in these organelles. However, how these proteins are transported to the mitochondria is unknown. Importantly, the initial steps of the mitochondrial protein import (targeting from cytosol to mitochondrial surface) often requires the involvement of preprotein-specific factors [191,192,202]. Thus, knowledge about the mechanisms of FGFRs’ transport into the mitochondria may facilitate in the design of therapeutic strategies aimed at the selective inhibition of FGFRs’ mitochondrial translocation.

## 6. Conclusions

FGFRs constitute a tightly regulated signaling system important for cell homeostasis. FGFRs are often overproduced or mutated in various tumors, and disrupted signaling facilitates carcinogenesis. Although recent years have brought about a large progress regarding understanding the FGFRs’ function, still little is known about the trafficking and intracellular role of these receptors. How do FGFRs reach the nucleus and mitochondria? Is mitochondrial trafficking specific only for FGFR1, or are other FGFRs are localized to this organelle as well? What are the other substrates of mitochondrial FGFRs? Which mitochondrial processes are affected by FGFRs? These and other questions remain to be answered. 

The internalization of RTKs occurs via numerous pathways. Up till now, FGFRs were reported to utilize three endocytic routes—CME, caveolae-mediated, and macropinocytosis. The involvement of other internalization pathways in FGFRs trafficking is unclear. As endocytic pathways regulate the duration and specificity of the propagated signals (and therefore cellular outcome), further studies are required for understanding the spatiotemporal control of FGFRs. 

FGFRs-mediated endocytosis is currently employed as a tool for the selective delivery of cytotoxic agents into cancer cells overproducing FGFRs in the ADC approach. In numerous cases, ADCs display side effects, which may be attributed to their partial mistargeting. An improvement of the selectivity and efficiency of drug delivery into the tumors may largely advance cancer treatment with ADCs. One of the possibilities is to improve the affinity of the targeting molecule within the ADCs. We have recently demonstrated that a high affinity promotes the internalization of engineered antibodies targeting FGFR1 in in vitro models [191,192]. The subsequent in vivo studies are missing. ADCs have to be delivered to the lysosomes for an efficient intracellular drug release. Multivalent targeting molecules inducing FGFRs clustering may improve the efficiency of endocytosis, engage other endocytic pathways, and improve the lysosomal delivery of ADCs in complex with FGFRs, as shown for other receptors [203,204,205,206]. 

One of the major challenges in the targeted therapies against cancers with aberrant FGFRs is the development of resistance against applied therapeutics, including ADCs [207]. A possible solution for this problem is a combination therapies involving molecules with different modes of action (e.g., TKI with anti-FGFs or anti-FGFRs antibodies, immune checkpoint inhibitors, or cytotoxic drugs) [184]. The cellular trafficking of FGFRs may be engaged in overcoming cancer cell resistance as well. Efficiently internalizing the cytotoxic conjugates of FGF2 while simultaneously attaching two cytotoxic drugs of a different mode of action displayed superior anticancer properties [208]. 

In summary, the cellular trafficking of FGFRs can be employed for the selective treatment of cancers with aberrant FGFRs, either alone or in combination with other strategies. However, further detailed studies on the cellular trafficking of FGFRs are urgently required. 

## Abbreviations

AB	acidic box
ADCs	antibody-drug conjugates
AP-2	adaptin-2 complex
CCPs	clathrin-coated pits
CIE	clathrin-independent endocytosis
CLIC	clathrin-independent carriers
CME	clathrin-mediated endocytosis
DAG	diacylglycerol
EMS	8p11 myeloproliferative syndrome
ERAD	ER-associated protein degradation
ESCRT-0	endosomal sorting complex required for transport-0
Esyt2	extended synaptotagmin-2
FGFs	fibroblast growth factors
FGFRs	fibroblast growth factor receptors
GPI	glycosylphosphatidylinositol
GRB2	growth factor receptor bound-2
HPV	human papilloma virus
Hrs	hepatocyte growth factor-regulated tyrosine kinase substrate
IL2R	interleukin-2 receptor
IM	mitochondrial inner membrane
IMS	mitochondrial intermembrane space
IP_3_	inositol triphosphate
LDCs	ligand-drug conjugates
MMAE	monomethyl auristatin E
mTOR	mammalian target of rapamycin
NCAM	neural cell adhesion molecule
OM	mitochondrial outer membrane
PAK1	p21-GTPase Activated Kinase
PDC	pyruvate dehydrogenase complex
PDHK1	pyruvate dehydrogenase kinase 1
PI3K	phosphoinositide 3-kinase
PKC	protein kinase C
PLCγ	phospholipase C-gamma
PSCs	pancreatic stellate cells
RSK1	p90 ribosomal S6 kinase 1
RSK2	p90 ribosomal S6 kinase 2
RTKs	receptor tyrosine kinases
S4	syndecan-4
SOS	son of sevenless
STAT	signal transducer and activator of transcription
TACC3	transforming acidic coiled-coil containing 3
TKIs	tyrosine kinase inhibitors
